# Increased ATP and ADO Overflow From Sympathetic Nerve Endings and Mesentery Endothelial Cells Plus Reduced Nitric Oxide Are Involved in Diabetic Neurovascular Dysfunction

**DOI:** 10.3389/fphar.2018.00546

**Published:** 2018-05-29

**Authors:** M. Verónica Donoso, M. Jesús Mascayano, Inés M. Poblete, J. Pablo Huidobro-Toro

**Affiliations:** Laboratorio de Farmacología de Nucleótidos, Departamento de Biología, Facultad de Química y Biología, Centro Desarrollo de Nanociencia y NanoTecnología, CEDENNA, Universidad de Santiago de Chile, Santiago, Chile

**Keywords:** Streptozotocin-induced diabetes, nucleotide release, extracellular adenosine, cultured endothelial cells, nitric oxide production

## Abstract

Since the mechanism of human diabetic peripheral neuropathy and vascular disease in type 1 diabetes mellitus remains unknown, we assessed whether sympathetic transmitter overflow is altered by this disease and associated to vascular dysfunction. Diabetes was induced by streptozotocin (STZ)-treatment and compared to vehicle-treated rats. Aliquots of the *ex vivo* perfused rat arterial mesenteric preparation, denuded of the endothelial layer, were collected to quantify analytically sympathetic nerve co-transmitters overflow secreted by the isolated mesenteries of both groups of rats. Noradrenaline (NA), neuropeptide tyrosine (NPY), and ATP/metabolites were detected before, during, and after electrical field stimulation (EFS, 20 Hz) of the nerve terminals surrounding the mesenteric artery. NA overflow was comparable in both groups; however, basal or EFS-secreted ir-NPY was 26% reduced (*p* < 0.05) in diabetics. Basal and EFS-evoked ATP and adenosine (ADO) overflow to the arterial mesentery perfusate increased twofold and was longer lasting in diabetics; purine tissue content was 37.8% increased (*p* < 0.05) in the mesenteries from STZ-treated group of rats. Perfusion of the arterial mesentery vascular territory with 100 μM ATP, 100 nM 2-MeSADP, or 1 μM UTP elicited vasodilator responses of the same magnitude in controls or diabetics, but the increase in luminally accessible NO was 60–70% lower in diabetics (*p* < 0.05). Moreover, the concentration–response curve elicited by two NO donors was displaced downwards (*p* < 0.01) in diabetic rats. Parallel studies using primary cultures of endothelial cells from the arterial mesentery vasculature revealed that mechanical stimulation induced a rise in extracellular nucleotides, which in the cells from diabetic rats was larger and longer-lasting when comparing the extracellular release of ATP and ADO values to those of vehicle-treated controls. A 5 min challenge with purinergic agonists elicited a cell media NO rise, which was reduced in the endothelial cells from diabetic rats. Present findings provide neurochemical support for the diabetes-induced neuropathy and show that mesenteric endothelial cells alterations in response to mechanical stimulation are compatible with the endothelial dysfunction related to vascular disease progress.

## Introduction

Diabetes mellitus is the most common endocrine disorder; predictions of the world health organization statistics indicate that 9% of the human population over 18 years will develop the disease (WHO, 2014). The current estimate is that 300 million people worldwide have elevated glycemia, a first indication of disease development (Mane et al., 2012). Diabetes mellitus type 1 is a syndrome with persistent medical complications; among the multiple consequences of the chronic disease are microcirculation failures, coronary artery disease, stroke, nephropathy, retinopathy, atherosclerosis, and peripheral diabetic neuropathy. It is not clear whether metabolic alterations lead to these vascular manifestations or whether these are separate entities that develop concurrently over the years of disease development.

In blood vessels, diabetes targets different cell types such as nerve terminals, smooth muscles, and/or the endothelium. In particular, sympathetic nerve endings are modified by diabetes altering either the tissue content or the release of sympathetic co-transmitters noradrenaline (NA), neuropeptide tyrosine (NPY), and ATP (Huidobro-Toro and Donoso, 2004). Regarding peripheral neuropathy, sympathetic neurotransmitters are known to be modified by diabetes, the effect depending largely on the timing of streptozotocin (STZ) treatment. In this regard, Kuncová et al. (2005) described that NPY decreased in heart ventricle six months after STZ administration; by nine months the immunoreactive NPY (ir-NPY) reduction was also extended to the atria. In parallel, heart NA increased during the first month following STZ administration, but decreased to 60% of control values by 12 months. Moreover, based on the responses to electrical field stimulation (EFS), and to the exogenous NA and ATP application to the mesenteric artery of STZ-treated rats, Ralevic et al. (1995) inferred the existence of disease-induced pre-junctional impairment. Furthermore, these authors concluded that experimental diabetes modified the smooth muscle reactivity to sympathetic co-transmitters or to an undetermined endothelium-derived factor, which is likely either nitric oxide (NO) or hyperpolarizing factor. Diabetes may well produce endothelium dysfunction as evidenced by reduced acetylcholine-induced vasodilatation in aortic segments or rat mesenteric micro vessels (Vallejo et al., 2000), an effect mediated mainly by NO, a free radical that interacts chemically with reactive oxygen species (ROS), reducing its biodisposition. Glycated proteins increase during disease development (AGE), a relevant clinical finding used as a predictor of disease severity. AGEs bind to one of its multiple cell surface receptors increasing ROS production which ultimately promotes endothelial cell (EC) dysfunction by several mechanisms (Funk et al., 2012).

Among the models used to induce experimental diabetes, the administration of STZ causes pancreas β-secretory cell destruction. STZ accumulates in pancreatic cells via glucose transporter 2; where it is metabolized intracellularly to a cytotoxic nitrosourea causing cell death by multiple mechanisms, resulting in loss of insulin production. Since at present there is no clear understanding of the mechanism involved in diabetes-induced neurovascular effector junction alterations, we now describe the effects of diabetes type 1 on the sympathetic innervation of the rat arterial mesenteric bed and its implications in the vascular manifestations of the disease’s symptoms. The classic McGregor (1965) *ex vivo* perfused rat mesenteric bed preparation is particularly useful as a physiological model, since it allows to study the release of sympathetic co-transmitters and other sensory transmitter molecules and associated tissue content; endothelium mediated vascular responses induced by conductance and resistance vessels where the role of NO as an endothelium vasodilator is physiologically preponderant. The activation of EC purinergic receptors is coupled with NO secretion (Buvinic et al., 2002), proposing that the release of ATP by ECs may cause NO-mediated vasodilatation. Previous reports from our laboratory used the rat mesentery as a vascular model to study biochemical and functional properties of the perivascular sympathetic nerve endings, particularly co-transmitter release (Donoso et al., 1997a, 2006a, 2012). This physiological preparation allows, on the one hand, assessing the functionality of this vascular bed by determining the changes in perfusion pressure, which indicates, the vascular tone of the arterial mesenteric territory (Buvinic and Huidobro-Toro, 2001). In addition, this preparation allows generating primary cultures of ECs as a relevant model to study ATP release (Donoso et al., 2018) and NO production in greater detail.

Present results reveal that STZ-induced diabetes modified the sympathetic nerve endings surrounding the mesenteric artery, reducing the outflow of ir-NPY while increasing ATP and adenosine (ADO) overflow elicited by EFS. In parallel studies, cultured ECs from diabetic rats showed a larger secretion of extracellular ATP to the cell media compared to control cells following mechanical stimulation. ATP receptor agonists elicited NO release in the arterial mesentery bed and also in cultured ECs. Moreover, both in mesentery perfusion as well as cultured ECs, purinergic agonists elicited NO production which was markedly blunted in diabetic rats. However, the vasodilator response of these agents was unaltered in the STZ-treated rats, an indication that other endothelial vasodilating factors must be at play. We consistently observed that vasodilatation induced by NO donors such as sodium nitroprusside (SNP) or S-nitroso-*N*-acetyl-D,L-penicillamine (SNAP) is reduced in STZ-diabetic rats, consonant with the proposal that NO bioavailability is decreased in diabetic animals. Modifications of sympathetic nerve endings plus EC alterations in diabetes are two determinant factors for the neuropathy and vascular complications associated to the disease.

## Materials and Methods

### Disease Model

Male Sprague Dawley rats (150–200 g) bred at the Animal Reproduction Facility of the Faculty of Biological Sciences of the P. Catholic University of Chile were used and animals were brought to local Universidad de Santiago Animal Laboratories. Experimental diabetes was induced by a single 65 mg/kg STZ dose, administered i.p. The drug was dissolved in 0.1 M citrate buffer, pH = 4.5; controls were injected with the same vehicle volume. Rats were fasted 6 h prior to the STZ administration; immediately thereafter, *ad libitum* food and water supplemented with 10% sucrose for 24 h was provided as described by Furman (2015). Animal safety protocols for the use of STZ in rodents were followed by the investigators and the Animal Laboratories personnel according to the guidelines of the Louisville University, KY, United States. Rat weight and plasma glucose were monitored once weekly after 6 h fasting (commercial Glucose kit, Accu-Chek, Roche). Protocols were performed 5–6 weeks after STZ treatment, when hyperglycemia levels were maintained fivefold elevated for at least 4 weeks.

Experiments were conducted in accordance with the American NIH guidelines for experimental animal use as detailed in the NIH Guide for the Care and Use of Laboratory Animals. The Ethical Committees of the Universidad de Santiago de Chile for the use of animals in biological research approved the specific protocols designed, and supervised our strict adherence to the subscribed guidelines as detailed in local protocol 198/2017. A total of 68 controls and 64 paired-matched STZ-treated rats were used throughout the project in the multiple protocols herein described, as will be specified in each of the experimental sections. To reduce the number of sacrificed animals, experiments were carefully planned for the conduction of several tests in a same rat tissue, when permissible.

### Perfusion of the Arterial Mesenteric Vasculature

The arterial mesenteric bed preparation was used as a bioassay to examine the release of sympathetic neurotransmitters and the vascular responses elicited by purinergic agents following the McGregor (1965) procedure with slight modifications described by Donoso et al. (2006a). To this aim, vehicle-treated controls and STZ-diabetic rats were anesthetized with ketamine (75 mg/kg) plus xylazine (5 mg/kg); a median laparotomy exposed the abdominal cavity; the superior mesenteric artery was dissected and cannulated *in situ* with plastic tubing to initiate perfusion of the mesentery vasculature with Tyrode buffer bubbled with a mixture of 95% O_2_/5% CO_2_ at 2 mL/min flow (37°C) through a peristaltic pump. The Tyrode solution composition was (mM): 118 NaCl, 5.4 KCl, 2.5 CaCl_2_, 1.2 KH_2_PO_4_, 1.2 MgSO_4_, 23.8 NaHCO_3_, 11.1 D-glucose. The whole cannulated mesenteric vascular bed including surrounding adipose tissue was carefully excised from the intestines and surrounding tissues and placed in a Petri dish maintained at 37°C with constant perfusion. Once the mesentery was removed from the abdomen, rats were sacrificed under deep anesthesia by pneumothorax plus aortic bleeding (Donoso et al., 2006a). The perfusion pressure of the mesentery vasculature was monitored continuously using a pressure transducer connected to a multichannel Grass polygraph. Pressure fluctuations were interpreted as changes in the resistance of the arterial mesenteric territory; changes in vascular pressure were quantified in mmHg.

#### EFS Elicited Sympathetic Neurotransmitter Overflow

To investigate the development of STZ-induced peripheral sympathetic neuropathy, the nerve terminals innervating the mesentery were subjected to EFS; the transmitters released to the vascular bed were collected to quantify the release of NA, ir-NPY, ATP, and related purines to the mesentery perfusate. These studies were performed in endothelium denuded mesenteric preparations to facilitate transmitter collection and avoid ATP/metabolites contamination from adjacent ECs. The endothelium was removed following a 55 s perfusion of Tyrode buffer supplemented with 0.1 % saponin (Figueroa et al., 2009); immediately thereafter, mesenteries were perfused with regular Tyrode buffer for a 60 min equilibration period to restore basal perfusion pressure. Platinum electrodes connected to a Grass S44 stimulator were placed surrounding the superior mesenteric artery; a 1 min EFS (20 Hz, 60 V, 1 ms duration) applied as described by Donoso et al. (2006a). This stimulation caused a rapid rise in the arterial mesentery perfusion pressure, denoting neuro-transmitter release. The elicited vasomotor response was quantified in each experiment and analyzed for statistical significance between vehicle-treated controls and STZ-treated rats. The arterial mesentery effluent was collected every 2 min in pre-chilled tubes, before, during, and after EFS. A 0.2 mL aliquot sample from the 4 mL collected per tube was filtered through 0.2 μm nylon membranes (Millex from Millipore, Cork, Ireland) and maintained in chilled tubes to assay ATP and metabolites. Aliquot samples were incubated with chloroacetaldehyde at 80°C for 40 min to synthesize the corresponding fluorescent etheno-purines. The reaction was stopped by placing the tubes on ice; samples were stored overnight at 4°C for HPLC separation and fluorescence detection quantifications (Buvinic et al., 2007). The remaining 3.8 mL of ach sample was kept on ice and supplemented with 17.5 pmol 3,4-dihydroxybenzylamine (internal standard for the NA quantification); the NA and NPY were retained in a small Sep-Pak C-18 reverse phase cartridge column (Waters, Milford, MA, United States) and eluted separately as detailed by Donoso et al. (2006a). Next, samples were evaporated to dryness and stored at -20°C. NA was quantified using an HPLC equipment coupled to an electrochemical detector, while NPY was determined as ir-NPY following radioimmunoanalysis. For this set of protocols, mesentery preparations derived from 18 vehicle-treated controls and 24 STZ-treated rats were examined.

Furthermore, to determine the tissue content of sympathetic co-transmitters the mesenteric bed vasculature from the same animals used for the co-transmitter overflow determinations protocols were, at the end of the protocol, homogenized in 7 mL of acid solution A: 0.1 N HCl, 0.1 mM EDTA, 0.01% sodium bisulfite, plus 100 pmol of 3,4-dihydroxybenzylamine, used as an internal standard for the HPLC chromatograms. As specified above, these determinations were performed in 18 vehicle-treated controls plus 24 STZ-treated rats. The homogenate was centrifuged at 126 g during 30 min at 4°C two times sequentially. A total of 1 mL of supernatant was neutralized with 2 mL of solution B: 0.2 M Na_2_HPO_4_.2H_2_0, 50 mM Na_2_CO_3_, and 0.1 mM EDTA. A total of 1 mL of this neutralized solution was concentrated by Sep-Pak and eluted separately for NA and ir-NPY determinations. A 0.2 mL neutralized solution was utilized for ATP and metabolites quantification as described in a previous paragraph; 0.1 mL of the neutralized solution was used for Bradford’s protein assay determinations.

#### Assessment of Vascular Reactivity by Purinoceptor Agonists and NO Donors

To monitor arterial vascular reactivity in control and STZ-treated rats, arterial mesenteric preparations with intact endothelium were pre-contracted with 50 μM NA to increase the tissue perfusion pressure prior to application of purinergic agents or NO donor perfusion for 5 min. 100 μM ATP, 100 nM 2-MeSADP, 1 μM UTP, or 30 μM BzATP were dissolved in Tyrode buffer containing 50 μM NA. To this aim, groups of mesenteric preparations derived from 10 vehicle-treated controls and eight STZ-treated rats were studied for vascular reactivity. Additional rat preparations were perfused with NO donors such as SNAP or SNP; concentration-response curve protocols were made with either agent. To this aim, groups of mesenteric preparations derived from nine vehicle-treated controls and seven STZ-treated rats were studied.

#### Quantification of Luminally Accessible NO Elicited by Purinergic Agonists

A parallel set of 15 vehicle-treated controls and 10 STZ-treated diabetic rats was aimed at quantifying the fraction of luminally accessible NO, following purinoceptor perfusion. In these protocols, care was taken to avoid pre-contraction with NA, since this procedure causes *per se* a significant NO rise (Boric et al., 1999). To this end, the mesentery vascular territory was perfused for 5 min with the purinoceptor agonists at the same concentrations used to evaluate changes in perfusion pressure; perfusate aliquots were collected in ice and rapidly sealed with parafilm for immediate sample analysis to determine the fraction of luminally accessible NO. Since the NO released to the perfusate is rapidly oxidized to nitrites, the method allows reducing nitrites to NO equivalents as reported by Boric et al. (1999). NO in the perfusate aliquots was quantified by chemiluminescence using a Sievers 280 NO analyzer, as detailed by Figueroa et al. (2009).

### Isolation and Harvesting of Primary ECs Cultures

The surgical procedure to isolate the rat mesentery was the same as that described in Section “Perfusion of the Arterial Mesenteric Vasculature”, except that these tissues had an intact endothelium. Mesenteries from control rats or STZ-treated animals were perfused with Tyrode buffer supplemented with 200 U/mL penicillin, 0.2 mg/mL streptomycin, and 0.5 μg/mL amphotericin-B at room temperature (20°C). The mesentery was incubated for 1 h in a beaker at 37°C under mild stirring in 5 mL of Tyrode buffer supplemented with the antibiotics plus 0.1% BSA and 2 mg/mL collagenase I. Thereafter, the cell suspension was centrifuged at 126 g at 4°C for 10 min to eliminate the mesentery adipose tissue and gross tissue debris. The cellular pellet was dissolved in M-199 medium plus antibiotics and next centrifuged at 126 g and 4°C for 5 min, re-suspended in 36 mL of medium 199 containing antibiotics and supplemented with: 20% fetal bovine serum (FBS) plus 20 μg/mL endothelial cell growth supplement (ECGS, Donoso et al., 2018). In addition, ECs isolated from the STZ-treated rats, were cultured in medium added with 25 mM glucose to maintain similar *in vivo* conditions. ECs were seeded in two 24 multi-well plates, and kept at 37°C until reaching ∼80% confluence an ideal time for our studies. 10 vehicle-treated rats and nine STZ-treated rats were used to examine the extracellular release of ATP/metabolites. In addition, six vehicle-treated rats and six STZ-treated rats were used to harvest ECs for the studies of purinoceptor-induced NO production; to this aim, ECs were plated in four separate wells (5.5 cm diameter).

#### Protocol Used to Determine Extracellular ATP/Metabolites Released Following Mechanical ECs Stimulation

To quantify the release of extracellular ATP/metabolites to the cell media, primary EC cultures were mechanically stimulated using the cell media displacement (CMD) procedure reported by Donoso et al. (2018). This protocol was adapted from the original report by Lazarowski et al. (1997). In addition, Romanello et al. (2001) or Hovater et al. (2008) also used this method to elicit extracellular nucleotide secretion from several cell types. One half (200 μL) of the cell media volume (400 μL) was gently pipetted up and down three consecutive times during less than 3 s, on the walls of each well using a P1000 micropipette. This procedure did not cause cell death as evidenced by lactate dehydrogenase (DHL) activity determinations in the cell media, or trypan blue uptake (data not shown). Cell media samples were collected following CMD to assay the extracellular ATP/metabolites released. Prior to the performance of the CMD protocol, ECs were incubated in Tyrode buffer supplemented with 10 mM HEPES at 37°C for 1 h; this precaution avoided interference from the regular cell medium, which contains 1 μM ATP. In the 24 multi-well plates, parallel protocols and corresponding controls were conducted. Some wells were maintained without mechanical stimulation and represent spontaneous basal secretion, other wells were subjected to CMD; 200 μL aliquots of the cell media were routinely collected 1, 3, 5, 10, and 15 min after the CMD procedure. These aliquots were placed on ice until chemical derivatization, to synthesize fluorescent purines as described in a previous Section “EFS Elicited Sympathetic Neurotransmitter Overflow”. After protocol completion, the wells were rinsed with Tyrode buffer, and the supernatants were discarded. The plate was placed at -20°C until Bradford protein determinations were performed in each well. The ECs used for these experiments derived for the animal specified in Section “Isolation and Harvesting of Primary EC Cultures.”

#### Purinoceptor Agonists Induced NO Production in ECs

The luminally accessible NO released by ATP and several purinergic receptor agonists with varying specificity for the P2Y and/or P2X receptors were examined. Cells were challenged during 5 min with ATP, 2-MeSADP, UTP, BzATP, at a time when a supernatant sample was retrieved rapidly sealed with parafilm and maintained in ice for immediate NO analysis as described in a previous Section “Quantification of Luminally Accessible NO Elicited by Purinergic Agonists.” The ECs used for these experiments derived for the animal specified in Section “Isolation and Harvesting of Primary EC Cultures.”.

### Drugs and Chemicals Used

Analytical grade solvents for Tyrode buffer, HPLC, and RIA analysis were purchased from Merck (Darmstadt, Germany). Adenosine 5′-triphosphate disodium salt hydrate (ATP); uridine 5′-triphosphate trisodium salt dehydrate (UTP); 2-(methylthio) adenosine 5′-diphosphate trisodium salt hydrate (2-MeSADP); 2′(3′)-O-(4-benzoylbenzoyl) adenosine 5′-triphosphate triethylammonium salt (BzATP); 3,4-dihydroxybenzylamine; (-)-norepinephrine (NA); streptozotocin (STZ): bovine serum albumin (BSA); Endothelial Cell Growth Supplement (ECGS); 4-(2-hydroxyethyl)piperazine-1-ethanesulfonic acid (HEPES); S-nitroso-*N*-acetyl-D,L-penicillamine (SNAP); sodium nitroprusside (SNP); saponin, were purchased from Sigma-Aldrich. NPY was obtained from Bachem. Media 199; penicillin; streptomycin plus amphotericin-B (Antibiotic-Antimycotic, 100×); fetal bovine serum (FBS), were obtained from Gibco. Collagenase type I, Worthington Biochemical Corporation.

### Data Presentation and Statistical Analysis

Sympathetic co-transmitter values are expressed in pmol/4 mL of each perfusate aliquot. NO determinations were quantified and expressed as either pmol NO/mL or as the Δ NO pmol/mL, a value attained by subtracting baseline NO efflux (a value attained prior to the agonist application) from that elicited by purinergic agonist application. Extracellular ATP/metabolites concentration values in the well cell media are expressed as pmol purines/mg protein. Vascular dilator responses induced by ATP and related purinergic agonists as well as NO donors are expressed as percent of the NA-induced pre-contraction.

For statistical analysis, the STATA 14.0 program, Texas, United States, was routinely used. Normal data distribution was determined by the Shapiro–Wilk method. To compare two sets of data, the Student’s *t*-test or Mann–Whitney test were used as appropriate, depending on the nature of the results distribution and convenience of parametric versus nonparametric analysis. To compare more than two sets of data, analysis of variance (ANOVA) or Kruskal–Wallis assays were used. The level of significance was defined as α < 0.05.

## Results

### STZ-Diabetes Rat Characterization

Fifteen days after the STZ dose, rats exhibited marked hyperglycemia which was maintained during the next four weeks. Glycemia increased at least 4.6-fold, reaching an average of 489.2 ± 18.3 mg/dL, in a group of 44 STZ-treated rats, compared with matched vehicle-treated controls which attained 106.2 ± 4.3 mg/dL (*n* = 34, *p* < 0.0001, Mann–Whitney test). In addition, STZ-treatment was associated with a substantial body weight loss compared to the same matched group of control rats (195.5 ± 10.8 g (*n* = 44), versus 371.5 ± 8.4 g (*n* = 34), *p* < 0.0001, unpaired Student’s *t*-test).

### Perfusion of the Arterial Mesenteric Vasculature

#### Sympathetic Neurochemicals Outflow Is Modified by STZ-Induced Diabetes

**FIGURE 1:**
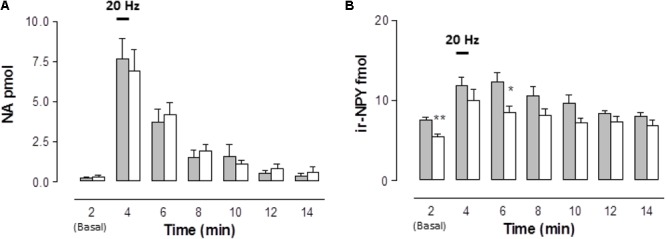
Time course of noradrenaline (NA) and neuropeptide tyrosine (NPY) released to the rat arterial mesenteric bed perfusates following EFS of sympathetic nerve terminals in endothelium-denuded preparations. Perfusate aliquots of the rat mesentery arterial vascular bed were collected to assay NA **(A)** and ir-NPY **(B)** released to the tissue perfusate before, during, and following EFS (symbolized as the 1 min 20 Hz bar). Grey columns indicate vehicle-treated controls (*n* = 16); open columns correspond to STZ-treated rats (*n* = 22 for NA determinations, while *n* = 24 for ir-NPY). Columns indicate the mean average values of the transmitter time course; bars indicate the SEM. ^∗^*p* < 0.05; ^∗∗^*p* < 0.01, is the Mann–Whitney test compared to its matched control at several times.

EFS evoked a rise in the overflow of NA, ir-NPY, and ATP/metabolites to the rat mesentery artery perfusate that represents a fraction of the total sympathetic co-transmitter released in endothelium-denuded mesenteries. Neither basal NA outflow nor those attained after EFS differed from those observed in the STZ-treated rats (**Figure 1A**). EFS induced NA outflow within 2 min and returned to basal values within 8-10 min (χ^2^(6,112) = 65.522, *p* = 0.0001, Kruskal–Wallis test); similar time courses and magnitudes of overflow were observed in the mesenteries from diabetic rats (10 min (χ^2^(6,154) = 56.698, *p* = 0.0001, Kruskal–Wallis test). Basal ir-NPY overflow was 26% reduced in the STZ-treated animals compared to the controls (Mann–Whitney test, **Figure 1B**). Time course analysis revealed that the rise in ir-NPY elicited by EFS (χ^2^(6,112) = 27.588, *p* = 0.0001, Kruskal–Wallis test) was lower in mesenteries from the STZ-treated rats than in controls (χ^2^(6,168) = 18.04, *p* = 0.0061, Kruskal–Wallis test, **Figure 1B**).

**FIGURE 2:**
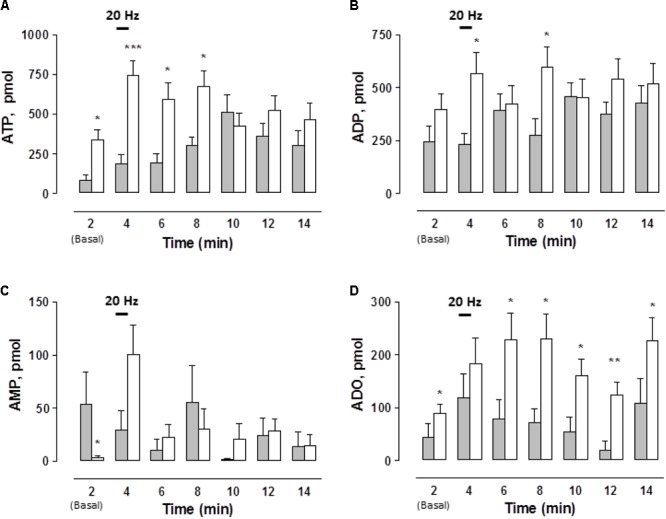
Time course of ATP and related purines detected in the rat arterial mesenteric bed perfusate upon EFS of the tissue nerve terminals in endothelium-denuded preparations. Perfusate aliquots from the rat arterial mesenteric vasculature were collected to assay ATP and related purine metabolites before, during, and after EFS (symbolized as the 1 min 20 Hz stimulation bar) of the tissue’s perivascular nerve terminals. **(A)** ATP, **(B)** ADP, **(C)** AMP, and **(D)** ADO. Gray columns indicate vehicle-treated controls (*n* = 10); open columns correspond to STZ-treated rats (*n* = 19). Columns indicate the mean average values of ATP and purine metabolites along the time course protocol; bars indicate the SEM.^∗^*p* < 0.05; ^∗∗^*p* < 0.01, and ^∗∗∗^*p* < 0.001, in case an ADP, unpaired Student’s *t*-test was applied, ATP, AMP, and ADO applied Mann–Whitney test as compared to matched control at the several times.

While the basal, spontaneous, ATP and ADO overflow values were increased in the STZ-treated rats, basal AMP outflow was significantly reduced (**Figure 2**). Following EFS, extracellular ATP and ADO increased above baseline, reaching two- to threefold higher values than the controls along its time course (**Figures 2A,D**). The pattern of the mesentery artery ATP overflow was different between vehicle-treated controls and the STZ-treated preparations. While in the controls the maximum ATP outflow was attained within 8 min, in the STZ-treated tissues the peak occurred during the second minute and was reasonably well maintained for the next 6 min (**Figure 2A**). Inversely, ADO peaked in the control preparations within 2 min and decreased thereafter, while in the mesenteries from diabetic rats the ADO rise remained elevated for at least 10 min (p < 0.05, **Figure 2D**). EFS did not significantly modify ADO outflow in the mesentery perfusate from control tissues (χ^2^(6,70) = 7.697, *p* = 0.2611, Kruskal–Wallis test) but in the STZ-treated rats, EFS significantly increased ADO overflow (χ^2^(6,133) = 14.143, *p* = 0.0281, Kruskal–Wallis test).

EFS did not modify the ADP overflow in the controls and in the mesenteries from the STZ-treated rats (**Figure 2B**). Likewise, no substantial changes were consistently attained when comparing AMP values between the controls and STZ-treated rats (**Figure 2C**). In control tissues, EFS did not significantly modify the time course of AMP outflow; in contrast, in STZ-treated rat mesenteries AMP levels were significantly increased by 10 min (χ^2^(6,133) = 17.379, *p* = 0.0080, Kruskal–Wallis test).

Considering the total outflow of purines secreted to the mesenteries following EFS, the sum of ATP plus ADP, AMP and ADO amounted to 2963.1 ± 556.4 (*n* = 10 controls), versus 4246.5 ± 568.1 pmol (*n* = 19 diabetics), evidencing a consistent 40% increase in the overflow of purines by diabetes. An additional control protocol was performed in 2 vehicle-treated rat preparations to assess whether the sole mesentery perfusion elicited *per se*, without EFS, variations in ATP outflow. Results monitored the mesentery outflow during 2 h without evidencing an increase in the ATP overflow, a likely indication that perfusion by itself did not cause cell damage as evidenced by the lack of ATP leakage alone the 2 h protocol course (data not shown).

The functional significance of transmitter co-released was evidenced as a rapid rise in the perfusion pressure of the mesentery vasculature, which peaked within 30 s after EFS delivery was maintained during the stimulation period and decayed to basal level in less than 30 s after EFS ended. We interpret the prompt rise in perfusion pressure as due to an intense constriction of the mesentery vascular territory induced by a synergic action of NA NPY plus ATP. In a group of vehicle-treated controls, the mean average rise in perfusion pressure over basal values was 92.8 ± 10 (*n* = 16) versus 87.8 ± 7.2 (*n* = 21) mmHg in the STZ-treated rats. Moreover, basal perfusion pressure did not differ between vehicle-treated controls (36.3 ± 5.2, *n* = 16) and a group of 21 STZ-treated rats (35.6 ± 2.6 mmHg).

To assess whether diabetes modified the synthesis and storage of sympathetic co-transmitters, we next examined the total tissue content of these chemicals in vehicle and STZ-treated rat mesenteries. While the NA content in the controls did not differ statistically from that observed in the STZ-treated rat tissues (88.5 ± 15.2 pmol/mg protein, *n* = 16, versus 113.7 ± 14.2 pmol/mg protein, *n* = 22), respectively. Regarding the mesentery ir-NPY content, the STZ-treated group of rats showed a significantly lower value than in the mesenteries from control rats (1.37 ± 0.1 (*n* = 24) versus 1.75 ± 0.2 pmol/mg protein (*n* = 16), *p* < 0.0423, unpaired Student’s *t*-test, respectively). This finding was consistent with the reduction in basal or EFS-induced ir-NPY outflow from these same mesenteries. Total tissue purines was greater in mesenteries from the STZ-treated animals (2381 ± 249 pmol/mg protein, *n* = 20) versus vehicle-treated controls (1718 ± 143 pmol/mg protein, *n* = 18, *p* < 0.0156, unpaired Student’s *t*-test), evidencing a 40% increase, similar to that observed in total purines overflow.

#### Diabetes Reduced Purinoceptor Mediated NO Overflow, but Did Not Affect Mesentery Vasodilation Elicited by Purinergic Agonists

**FIGURE 3:**
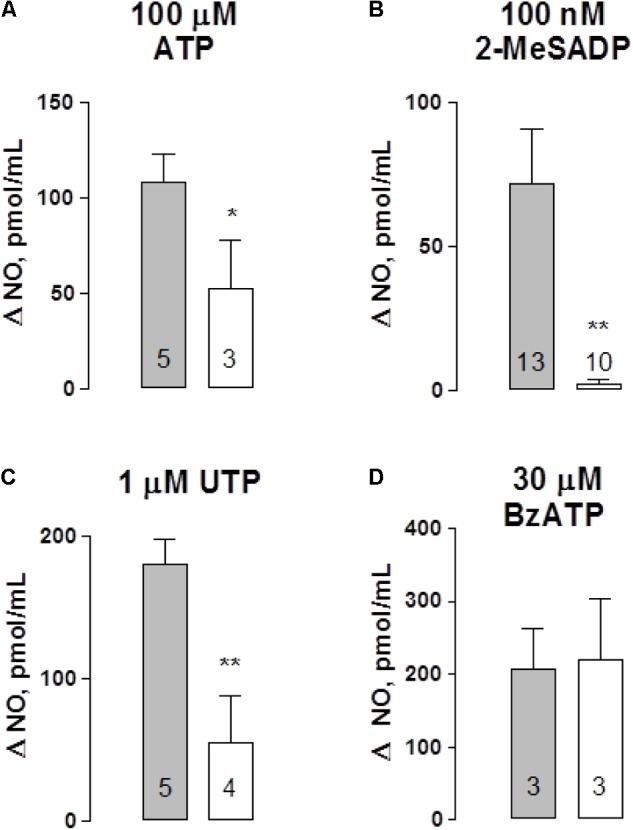
Nitric oxide (NO) detection in perfusates of the rat arterial mesenteric vasculature following purinergic agonist administration. Purinergic agonists for several receptor subtypes were perfused to the mesenteric preparation; perfusate aliquots were collected to quantify NO. Perfusion with: **(A)** ATP, **(B)** 2-MeSADP, **(C)** UTP, and **(D)** BzATP. Results are expressed as Δ NO pmol/mL obtained by subtracting basal values from those elicited following nucleotide application to the mesentery perfusate. Gray columns indicate vehicle-treated controls; open columns correspond to the matched STZ-treated diabetic rats. Columns indicate mean average values; bars represent the S.E.M. Numbers inside the columns represent the rat preparations used in these determinations. ^∗^*p* < 0.05; ^∗∗^*p* < 0.01; in the case of 2-MeSADP, the Mann–Whitney test, ATP, and UTP unpaired Student’s *t*-test were applied.

To link ATP secretion to the NO-mediated vasodilatation in this vascular bed we set to assess whether perfused ATP and related purinergic agonists elicited NO production in mesentery artery perfusates. Basal NO was detected luminally; no difference in NO values were observed when comparing tissues from vehicle-treated controls versus STZ-treated rats (111.7 ± 9.4 pmol/mL, *n* = 15 versus 111.1 ± 7.4 pmol/mL, *n* = 10, respectively); an indication that the sole buffer perfusion of this vascular territory permitted detecting a baseline of luminally accessible NO as reported by Boric et al. (1999). Upon tissue perfusion with 100 μM ATP, a rise in the perfusate NO was evidenced; the NO rise over basal values (Δ NO, expressed as pmol/mL) amounted to 108 ± 15 pmol NO/mL (*n* = 5), a value that was reduced 48.5% in the STZ-treated mesenteries (*p* < 0.05, **Figure 3A**). However, the magnitude of the ATP-induced vasodilatation was not modified in these two groups of rats (**Table 1**). Likewise, perfusion with either 100 nM 2-MeSADP or 1 μM UTP also elicited a significant NO rise, reaching 72 ± 19 and 180 ± 18 pmol NO/mL respectively, values which were reduced by 96.7 and 70% respectively, in STZ-treated rat tissues (*p* < 0.01, **Figures 3B,C**). As observed with ATP, the reduction in the perfusate accessible NO elicited by 2-MeADP or UTP was not associated with a proportionally decreased vasodilator response in the STZ-treated rats (**Table 1**). In contrast with the other purinoceptor agonists examined, BzATP increased the mesentery artery perfusion pressure, an indication of a robust vasomotor effect. The 30 μM BzATP-induced vasocontractile response was similar to that attained in diabetic rat mesenteries (**Table 1**). Moreover, 30 μM BzATP, a purported P2X1 and P2X7 receptor agonist, elicited a similar rise of NO in controls and STZ-treated rats (**Figure 3D**), an effect probably associated with the intense tissue vasoconstriction elicited by this purinoceptor ligand.

**Table 1:** Dilatation of the rat arterial mesenteric bed territory induced by perfusion with ATP or selective purinergic agonists.

	Controls	STZ-treated rats
**Vasodilatation (%, x ± SEM)**		
100 μM ATP	63.4 ± 4.3 (*n* = 5)	60.6 ± 9.1 (*n* = 4)
100 nM 2-MeSADP	85.4 ± 6.1 (*n* = 5)	87.1 ± 3.6 (*n* = 4)
1 μM UTP	22.1 ± 6.7 (*n* = 5)	5.5 ± 5.5 (*n* = 3)
30 μM BzATP	–89.7 ± 14.4 (*n* = 5)	–114.5 ± 9.2 (*n* = 5)

*Negative vasodilation indicates vasocontraction induced by BzATP.*

**FIGURE 4:**
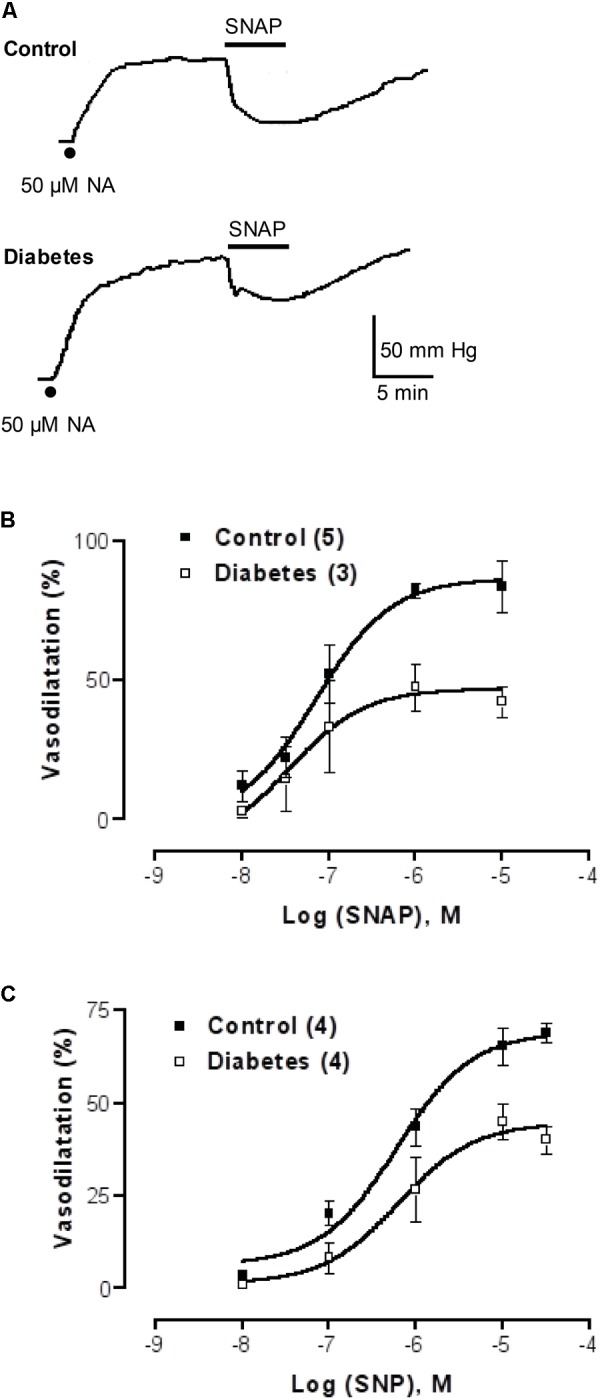
NO donor concentration-response curves in mesenteries from control and diabetic rats. **(A)** Representative recordings of 1 μM SNAP-induced vasodilatation in the rat arterial mesenteric bed of a control and a parallel protocol performed on a diabetic rat mesentery. Filled dots represent mesentery pre-contraction with 50 μM NA prior to the addition of NO donors. **(B,C)** show SNAP and SNP concentration-response curves, respectively, in mesenteries from control and diabetic rats. Symbols indicate mean average values; bars represent the SEM; filled squares refer to controls while the open squares represent the mesenteries from STZ-treated rats. Both the SNAP and SNP curves proved statistical significance when comparing control versus diabetic animals.

To further expand these observations, and to examine whether the difference between the mesentery artery vasodilatation and luminally accessible NO was extended to other vasodilators not related to purinoceptor signaling, we tested whether NO donors such as SNAP or SNP behaved similarly. Perfusion with SNAP or SNP induced concentration-dependent vasodilator responses which were significantly attenuated in the STZ-treated rats compared to the parallel controls (**Figure 4** and accompanying representative polygraph tracings). The concentration-response curves of the two NO donors examined were significantly reduced in the STZ-treated rats, SNAP (F(1,31) = 8.34, *p* = 0.0075, **Figure 4B**) and the SNP (F(1,25) = 14.3, *p* = 0.0009, **Figure 4C**) as compared to their corresponding control group of rats.

### Studies With ECs

#### ECs From Diabetic Rats Secrete More ATP and ADO to the Cell Media Following CMD Than Controls

**FIGURE 5:**
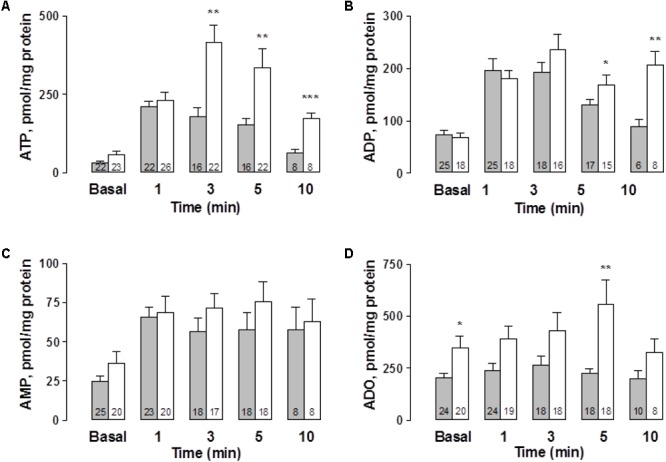
Time course of extracellular ATP release and its metabolites following cell media displacement from primary rat mesentery ECs culture. Cell media aliquots from wells not exposed to CMD (basal values), or parallel aliquots from wells 1, 3, 5, and 10 min after CMD, were collected from vehicle-treated controls (gray columns) or ECs derived from mesenteries of STZ-treated animals (open columns). **(A)** ATP, **(B)** ADP, **(C)** AMP, and **(D)** ADO. Columns indicate mean average values; bars refer to the SEM. Numbers at the bottom of each column indicate replicas of each purine detection assay. ^∗^*p* < 0.05; ^∗∗^*p* < 0.01, ^∗∗∗^p < 0.001, compare the release of these purines in cell cultures from controls and STZ-treated rats. ATP and ADO values were compared using the Mann–Whitney test, while for the ADP comparisons the unpaired Student’s *t*-test was used.

To further extend the notion that the vasodilator effect of ATP is associated with NO production, we next examined, using cultured ECs from the rat mesentery, whether ATP secretion elicited by mechanical stimuli such as CMD (Donoso et al., 2018) is associated with NO production. This procedure somehow mimics shear stress forces detected by ECs which respond eliciting extracellular ATP secretion and thereafter NO production. To this end, we prepared ECs and evaluated both the spontaneous ATP overflow as well as the nucleotide secretion elicited by the CMD procedure in ECs from vehicle- and STZ-treated rat groups. Basal extracellular nucleotides, which reflect the spontaneous purine secretory pool of ECs, each attained a different basal level, a likely indication of active cellular metabolism of extracellular purines. ATP and AMP baseline values in the control groups are the lowest (**Figures 5A,C**), while the highest are ADP and ADO (**Figures 5B,D**). Extracellular ADO is 8.2 times higher than AMP and 2.8 times higher than ADP. Basal nucleotide values in ECs from STZ-treated rats were about the same as those observed in the vehicle-treated controls, except for ADO, which was 70% higher than in the vehicle-treated control groups (*p* < 0.05, **Figure 5D**). At 1 min after the CMD, ATP and metabolite values increased over the basal values. While the ECs from the vehicle-treated group reached a maximum level of extracellular ATP within one min and decayed thereafter, the cells from the diabetes groups reached higher values (*p* < 0.01) and did not reach baseline within 10 min (**Figure 5A**). Statistical analysis of ATP secretion showed that ATP increase over the basal levels was significantly different from the basal values of the controls (χ^2^ (4, 84) = 51.890, *p* = 0.0001, Kruskal–Wallis test) and the STZ-treated rats group of cells (χ^2^(4,101) = 54.205, *p* = 0.0001, Kruskal-Wallis test). Similarly, extracellular ADP doubled 1 min after the stimulus in both cell groups (**Figure 5B**). In the vehicle-treated group, ADP concentrations rose from 73 ± 9.8 (*n* = 25) to 196 ± 23 pmol ADP/mg protein (*n* = 25) while in the STZ-treated group the values increased from 68.8 ± 8.6 (*n* = 18) to 180 ± 15 pmol ADP/mg protein (*n* = 18). ADP values in the STZ-treated group remained elevated over the controls and did not reach baseline within 10 min (*p* < 0.01). Extracellular AMP analysis showed an almost two- to threefold increase over basal values in ECs from both groups, which did not return to basal values within 10 min (χ^2^(4,92) = 28.32, *p* = 0.0001, Kruskal–Wallis test for controls, and χ^2^(4,83) = 10.22, *p* = 0.0369, for cell from STZ-treated rats, **Figure 5C**). Extracellular ADO, whose basal value was 1.7 higher in the cells from the STZ-treated rats (*p* < 0.05), was essentially not modified by the CMD maneuver in control ECs, while in the ECs from the STZ-treated group, ADO values remained above the basal level, reaching a 1.6-fold higher value within 5 min *(p* < 0.05, **Figure 5D**); and decayed to basal values thereafter.

#### Reduced NO Secretion to the Cell Media Elicited by Purinoceptor Ligands in ECs From Diabetic Rats

To investigate whether ECs release NO upon exposure to purinoceptor agonists as was observed in the perfusion of the arterial mesenteric vasculature, we challenged ECs with ATP, 2-MeSADP, UTP or BzATP for 5 min. We observed that basal NO value was markedly increased following nucleotide addition. ECs incubated with 100 μM ATP increased NO by 60% (*n* = 6, **Table 2**). In contrast, 100 μM ATP addition to ECs from the diabetic animals caused a lower rise, reaching only 17% of the NO detected in the basal condition (**Table 2**). Similarly, application of 100 nM 2-MeSADP or 1 μM UTP elicited a significant rise in NO production, reaching values almost similar to that elicited by ATP; however, NO values were significantly reduced in ECs derived from STZ-treated rats (**Table 2**). Application of 30 μM BzATP also increased NO production in ECs from control rats, eliciting an increase similar to that attained with the other purinoceptor agonists, in the ECs from the STZ-treated rats, but the rise in NO was similar to that observed in the controls (**Table 2**), an indication that other receptors must be involved in the effects of this receptor. In addition, the basal NO from vehicle-treated controls was 119.5 ± 11.0 pmol/mL (*n* = 20), a value that was not different from that observed in ECs derived from diabetic animals (104.3 ± 11.9 pmol/mL, *n* = 23). These results are summarized in **Table 2**.

**Table 2:** NO production by ATP and related purinoceptor agonists in primary EC cultures derived from the rat arterial mesenteric bed of vehicle-treated controls and STZ-treated rats.

	Controls				STZ-treated rats			
	Basal	Nucleotide stimulated	Increase (%)	*n*	Basal	Nucleotide stimulated	Increase (%)	*n*
**NO pmol/mL (x ± SEM)**				
100 μM ATP	132.2 ± 29.3	198.3 ± 33.6	59.7 ± 12.2	6	100.8 ± 26.4	119.3 ± 20.3^a^	16.5 ± 5.2^bb^	6
100 nM 2-MeSADP	118.6 ± 22.2	201.8 ± 19.4*	81.6 ± 17.8	5	91.8 ± 27.5	122.8 ± 25.9^aa^	59.9 ± 22.3	6
1 μM UTP	120.4 ± 17.5	202.8 ± 29.5*	73.8 ± 20.3	5	93.7 ± 22.4	120.3 ± 16.9^a^	14.7 ± 2.2^bb^	6
30 μM BzATP	100.5 ± 12.9	160.3 ± 34.8	65.9 ± 36.4	4	128.7 ± 22.4	203.8 ± 28.9*	66.9 ± 15.6	6

*^∗^*p* < 0.05 nucleotide stimulated versus basal, Mann–Whitney test for 2-MeSADP, unpaired Student’s *t*-test for UTP, BzATP. ^*a*^*p* < 0.05, ^*aa*^p < 0.01, nucleotide stimulated controls versus STZ-treatment rats, unpaired Student’s *t*-test for ATP and UTP, Mann–Whitney test for 2-MeSADP; ^*bb*^*p* < 0.01 % controls increase (%) versus STZ-treatment rats, unpaired Student’s *t*-test.*

## Discussion

The present results show that experimental type 1 diabetes induced significant biochemical alterations of the sympathetic neuroeffector junction innervating the rat arterial mesenteric bed. Observations derived from mesenteries denuded of the endothelium layer; a preparation particularly predisposed to quantify the secretion of sympathetic co-transmitters with minimal endothelium interferences, or a minor component derived from the vascular smooth muscles as demonstrated by the studies of Bodin et al. (1991). In agreement with our aims, we compared results between matched vehicle-treated controls versus STZ-treated rats, a well-characterized laboratory model of human type 1 disease. Animals were routinely sacrificed for experimental purposes 37–40 days following the STZ dose. Careful control of the dosing period is relevant since we are aware that diabetes has a progressive nature, therefore the symptoms vary depending on its development. At 12–15 days after STZ, a sustained 4.6-fold glycemia increase was attained which lasted for the next 4 weeks of the experiments. In addition, rats became skinnier, though motility was not impaired. Present results consistently demonstrate that STZ-induced diabetes elicited significant alterations of sympathetic neurotransmitter biogenesis. In addition similar endothelium impairment was observed both in the *ex vivo* perfusion studies as well as in the primary EC cultures, an indication that the experimental model is reproducible allowing extrapolation of whole animal studies to EC cultures.

We choose the rat arterial mesenteric bed, a tissue used to study biochemical and functional properties of the perivascular sympathetic nerve endings, a preparation that we have extensively used in the past to characterize sympathetic co-transmitter overflow (Donoso et al., 1997a,b, 2006a,b, 2012). This preparation allowed recording purinergic agonist- induced vasodilatation by determining changes in perfusion pressure over time and estimating the fraction of luminally accessible NO elicited by purinergic ligands and NO donors. By using endothelium-denuded preparations, the release of ATP/metabolites is substantially less contaminated by the eventual release of endothelium-derived purines. We did not observe diabetes-induced changes in the release of NA provoked by EFS, a finding consistent with the conclusions of Johansen et al. (2013). In all the preparations examined, we observed a reduction in ir-NPY overflow following EFS or in basal overflow levels, as well as in the ir-NPY tissue levels in agreement with Milner et al. (1992), who showed ir-NPY reduction in the rat paravertebral sympathetic nerves. Since NPY acts in vascular neuroeffector junctions as a co-transmitter increasing the excitatory NA and ATP motor responses (Donoso et al., 1997a,b, 2004, 2006b; Racchi et al., 1999), this data allows to infer that perhaps the vasomotor response evoked by EFS might be reduced. Since only a modest 5% reduction was observed following EFS-evoked vasomotor responses; we deduced that the ATP and particularly ADO overflow must be substantially increased in diabetes, a result paralleled by a 1.4-fold increase of total purine found both in the mesentery overflow and tissue content. The latter finding is an indication that ATP synthesis and metabolism is likely increased in diabetes, data consistent with other studies and particularly with the notion of increased ROS production in diabetics as will be discussed in the next paragraphs. It is plausible to propose that the lack of differences in vasomotor responses elicited by EFS between controls and diabetic rats results from a balance between the reduced overflow of ir-NPY compensated by a larger overflow of ATP and ADO. Moreover, it is relevant to keep in mind that the increased ATP overflow over baseline values in the *ex vivo* preparation was paralleled by the values obtained in cultured ECs from the STZ-treated animals maintained in high glucose to closely mimic the diabetic animal’s hyperglycemia.

The basal and EFS-induced ATP and ADO overflow increased in STZ-treated animals, as well as the overflow from EC cultured from STZ-treated rats indicates that during the course of the disease purine metabolism changes. The increase in the ATP concentration in the extracellular space depends on its release and/or its degradation rate, both mechanisms may operate concurrently. The increase in the basal ATP level and that induced by stimulation is an indication that the extracellular degradation of ATP/ADP was reduced, as was reported by Costa et al. (2009), in rat retinal cell cultures in high glucose, or in encephalic membranes from hyperglycemic zebrafish (Capiotti et al., 2016). In addition, we infer that the diabetes-increased ATP released was similar to that reported in platelets from diabetic patients stimulated with thrombin (Michno et al., 2007). We are aware that future experiments planned specifically to address this issue are necessary; we are working to solve this caveat.

Various possible mechanisms may be invoked to account for the larger extracellular ADO levels determined in basal or stimulated nerve endings or in primary cultures of ECs particularly from STZ-diabetes rats. (a) ADO transporter expression may be reduced in diabetes a finding that accounts, at least in part, for the increased extracellular ADO levels found during disease progression. HUVEC cells from gestational diabetes or HUVEC cells grown experimentally in high glucose media reduce ADO transporter expression (Sobrevía et al., 1994; Montecinos et al., 2000), providing a direct link to the relevance of our observations. In addition, several studies concur to indicate that higher ADO levels are observed in diabetics either because of a lesser ENT-1 expression, the physiologically most relevant equilibrative nucleoside transporter characterized by its massive ADO mobilization (Pawelczyk et al., 2003). (b) An increased CD 73 activity or expression presumably increases AMP to ADO metabolism, accounting for the increased ADO levels in diabetes. In this context, Oyarzún et al. (2015) found an increase in the expression of CD73 in STZ-diabetic rat kidneys. Likewise, Sakowicz and Pawelczyk (2002) reported an increase in CD73 activity in the heart, liver, and kidneys of STZ-induced diabetic rats. (c) Reduced ADO metabolism by ADO deaminase also favors increased ADO levels. Encephalic membranes from hyperglycemic zebrafish exhibit a reduced expression of ADO deaminase (Capiotti et al., 2016), a finding that could be relevant to the current results.

ADO as a vasorelaxant agent acts apparently by a dual mechanism, since it dilates smooth muscles, an effect mediated by vascular A2A and A2B receptors, both localized in smooth muscle cells (Hiley et al., 1995; Prentice et al., 1997). Moreover, ADO also produces NO in ECs, an effect mediated by A1 and A2A receptors (Ray and Marshall, 2006). Furthermore, ADO increases cytokinin release, has anti-inflammatory and anti-proliferative properties and through presynaptic receptors of sympathetic nerve terminals reduces co-transmitter release (Donoso et al., 2006a). Therefore, we may no longer think of ADO as a terminal ATP metabolite devoid of biological significance. As discussed, ADO has multiple roles in the blood vessel wall, causing a compounded response which may be implicated in diabetes and its vascular consequences along the progression of the disease. This result leads us to hypothesize that the rise in ADO levels over the baseline may be functionally related to reduce local tissue inflammation and increase blood flow in an attempt to compensate the ongoing diabetes-induced increased ATP outflow from nerve endings as well as the ECs.

Since diabetes is frequently associated with endothelial dysfunction, and to determine whether this alteration prevails in the STZ-disease model, we quantified EC NO production by both the *ex vivo* mesenteric preparation and the isolated EC cultures. Extracellular ATP secretion to the mesentery circulation confirms the double role of nucleotides in vascular beds (Burnstock, 2008; Khakh and Burnstock, 2009). On one hand, ATP released from nerve terminals, as is the case of the present study, reaches adjacent vascular smooth muscle cells to elicit vasomotor responses probably mediated by P2X1 and P2X7 receptors. In addition, part of the neuronal secreted ATP plus a fraction derived from ECs is probably a signal through autocrine P2Y1 and P2Y2 receptors, leading to NO synthesis (Buvinic et al., 2002) or the release of endothelium-derived hyperpolarizing factor (EDHF) or other endogenous dilators of the mesenteric vessels, such as nitroxyl (HNO), which elicits smooth muscle dilatation (Andrews et al., 2009; Rieg et al., 2011; Yin et al., 2017). The luminally accessible NO released following P2Y receptor activation was reduced in STZ-treated rats compared to the matched vehicle-treated controls. Moreover, the ATP and more selective P2Y receptor agonists elicited vasodilator responses were not modified in the STZ-treated animals, in spite of the luminally accessible perfusate NO was reduced by the pathology. Therefore, to account for these findings, we hypothesize that perhaps excessive ROS production in diabetes, due to an intensified diabetic metabolic rate, reacts with endothelial-derived NO, trapping and chemically modifying this potent endogenous dilator as first described by Paolocci et al. (2001) and later complemented by Funk et al. (2012). In support of this contention, the levels of ROS and expression of NAPDPH oxidase 2 mRNA, is increased almost 4-fold in the mesenteric artery of diabetic rats as compared to the femoral artery from the same rats, which remained unaltered by the disease, illustrating tissue specific alterations (Tare et al., 2017). Likewise, superoxide generation in the rat mesenteric artery is also increased by diabetes (Tare et al., 2017). Based on these premises, the reduced NO donor-induced vasodilatation observed in the STZ-treated rats, which is totally dependent on NO availability, confirms our proposal. As a consequence, we propose that ECs from diabetic rats, which likely produce as much NO as control individuals, suffer from endothelial dysfunction due to a lesser functional NO bioavailability. Since the magnitude of the vasodilator response did not change in diabetics, this observation allowed us to infer that perhaps the role of EDHF or HNO is predominant in the purinoceptor-induced mesenteric relaxations.

BzATP, a P2X1 and P2X7 receptor agonists consistently elicited NO release from endothelial cells either in the perfused mesenteries protocols or when the nucleotide was added to cultured ECs. This nucleotide ligand caused vasomotor responses, confirming the dual extracellular ATP signaling role. Likewise, in macrophages Sperlágh et al. (1998) evidenced a larger ATP-induced NO production by lipopolysaccharide, a modulator of the P2X7 receptor. At present the role of P2X7 receptors in the arterial mesenteric bed or ECs remains enigmatic; since P2X7 receptor knock out mice show that the vasomotor effect of BzATP on the mesenteric arteriolar myogenic tone was not modified (Kauffenstein et al., 2016), a probable indication of the predominant P2X1 receptor activity in the arteriolar vasomotor tone of this tissue.

In conclusion, the present results highlight that within 5–6 weeks of STZ-induced diabetes, sympathetic nerve terminal alterations are relevant and are related to a reduced ir-NPY overflow plus increases in ATP and ADO outflow in the sympathetic nerve endings of the rat arterial mesentery bed. Moreover, we report a reduced luminally accessible NO overflow in the STZ-treated rats elicited by purinoceptor activation or NO donors induce vasodilatation of this vascular territory. Furthermore, we consistently observed increased extracellular ATP and ADO levels in EC culture media from the STZ-treated rats upon the CMD procedure. Moreover, NO production elicited by purinoceptor agonists in ECs from diabetic rats was reduced as compared to matched ECs from vehicle-treated rats. Altogether, the present findings add neurochemical evidence for peripheral diabetes-induced neuropathy and the disease-associated vascular dysfunction.

## Author Contributions

MVD designed and executed a substantial part of the protocols and supervised MJM and wrote a preliminary manuscript draft. MJM performed the endothelium denuded mesenteric perfusion studies under MVD’s guidance as part of an undergraduate dissertation. IP performed NO determination, while JPH-T assisted designing protocols, provided experimental group supervision, data interpretation, and grant funding, and edited the multiple manuscript versions.

## Conflict of Interest Statement

The authors declare that the research was conducted in the absence of any commercial or financial relationships that could be construed as a potential conflict of interest.
